# The state of antibiotic stewardship programs in 2021: The perspective of an experienced steward

**DOI:** 10.1017/ash.2021.180

**Published:** 2021-08-05

**Authors:** Tamar F. Barlam

**Affiliations:** Section of Infectious Diseases, Department of Medicine, Boston Medical Center, Boston, Massachusetts

**Keywords:** Antibiotic Stewardship Programs, COVID-19, antibiotic stewardship interventions

## Abstract

Recognition of antibiotic stewardship programs (ASPs) as essential components of quality health care has dramatically increased in the past decade. The value of ASPs has been further reinforced during the coronavirus disease 2019 (COVID-19) pandemic because these programs were instrumental in monitoring antibiotic use, assessing emerging COVID-19 therapies, and coordinating implementation of monoclonal antibody infusions and vaccinations. ASPs are now required across hospital settings as a condition of participation for the Centers for Medicare and Medicaid Services and for accreditation by The Joint Commission. In the 2019 National Healthcare Safety Network annual survey, almost 89% of hospitals met the Seven Core Elements for ASPs defined by the Centers for Disease Control and Prevention. More than 61% of programs were co-led by physicians and pharmacists, evidence of the leadership role of both groups. ASPs employ many strategies to improve prescribing. Core interventions of preauthorization for targeted antibiotics, prospective audit and feedback, and development of local treatment guidelines have been supplemented with numerous emerging strategies. Diagnostic stewardship, optimizing duration of therapy, promoting appropriate conversion from intravenous to oral therapy, monitoring at transitions of care and hospital discharge, implementing stewardship initiatives in the outpatient setting, and increasing use of telemedicine are approaches being adopted across hospital settings. As a core function for medical facilities, ASP leaders must ensure that antibiotic use and ASP interventions promote optimal and equitable care. The urgency of success becomes progressively greater as complex patterns of antibiotic resistance continue to emerge, exacerbated by unpredictable factors such as a worldwide pandemic.

Antibiotic stewardship programs (ASPs) have existed for more than half a century, but their recognition as an essential component of quality health care has dramatically increased in the past decade. To best understand the state of stewardship today, it is important to reflect on the factors that have contributed to that recognition, the current penetration of antibiotic stewardship within health care, innovative approaches gaining traction in the field, and possible future priorities.

Historically, ASPs have often been viewed as antibiotic restriction programs, measured by the magnitude of cost savings rather than the value of improved patient outcomes, lowered risk of treatment failure, and the potential to ameliorate the emergence of antibiotic resistance.^1,2^ When the term antibiotic stewardship was coined,^3^ it was immediately embraced because it more accurately reflected the central goals of ASPs to support appropriate antibiotic use: the right drug, at the right dose, for the right reason and the right duration.

ASPs have been plagued by inadequate resources and limited buy-in from hospital leadership and clinician prescribers.^4,5^ Hospital administrators eliminated programs when they mistakenly believed prescribing improvements would be sustained without further support, only to see a marked rebound in inappropriate prescribing and costs to the hospital.^6^ The increasingly urgent crisis of antibiotic resistance,^7^ as well as the priority to improve healthcare quality and reduce adverse, highly morbid events such as *Clostridioides difficile* colitis, have shifted perceptions of ASPs.^8^ Notably, in 2014, President Barack Obama issued an executive order to combat antibiotic-resistant bacteria that clearly included antibiotic stewardship as an important strategy. The Presidential Advisory Council on Combating Antibiotic-Resistant Bacteria (PACCARB) developed the National Action Plan for Combating Antibiotic-Resistant Bacteria,^9^ which called for universal implementation of ASPs in acute-care hospitals.

Advocacy by PACCARB, professional societies, and public health experts have borne fruit. For accreditation, The Joint Commission (TJC) added a new medication management standard for hospitals, including critical-access hospitals, and nursing care centers^10^ largely structured on the Core Elements for Hospital ASPs defined by the Centers for Disease Control and Prevention (CDC),^11^ a leader in combating antibiotic resistance and supporting antibiotic stewardship.^12^ In January 2020, the TJC added a requirement for all TJC-accredited ambulatory healthcare organizations to identify an antibiotic stewardship leader and establish an annual goal with evidence-based guidelines, educational resources for clinical staff, and analysis and reporting of data related to that antibiotic stewardship goal.^13^ Importantly, the Centers for Medicare and Medicaid Services (CMS) issued a Condition of Participation (CoP) that every participating hospital as of March 30, 2020, including critical-access hospitals, have an active facility-wide ASP to reduce inappropriate antibiotic use and antibiotic resistance with designated leaders to guide and oversee the programs.^14^ These are major victories for the advancement of ASPs from being perceived as optional programs at risk of elimination to mandatory programs central to quality care and public health.

## Antibiotic stewardship programs and the COVID-19 pandemic

Over the past year, the coronavirus disease 2019 (COVID-19) pandemic has reinforced the importance of ASPs and has broadened the appreciation of the different ways that ASPs are essential to the functioning of healthcare facilities.^15^ COVID-19 is a new disease with constantly evolving knowledge and information. Patients presented with severe respiratory disease, and often had leukocytosis and elevated procalcitonin and inflammatory markers. Evaluation and bedside examinations were challenging; radiography and diagnostic procedures were minimized when possible. The default, particularly early in the pandemic, was to treat with broad-spectrum antibiotics for possible secondary bacterial infection. Patients with prolonged hospitalizations continued to have antibiotic exposure. Antibiotic use tracked with the number of COVID-19 patients hospitalized; hospitals in the highest quartiles of COVID-19 cases showed greater increases in antibiotic use.^16^ Not surprisingly, increases in antibiotic resistance were also noted. Nosocomial pathogens identified in hospitalized patients demonstrated a 42% increase in methicillin-resistant *Staphylococcus aureus* and a 134% increase in extended-spectrum β-lactamase–producing gram-negative bacteria.^16^

As the pandemic progressed, clinicians learned from experience and from numerous studies reporting that coinfection with COVID-19 and bacterial or fungal infection at presentation were relatively infrequent,^17,18^ but inappropriate antibiotic use remained a significant issue at many facilities.^19^ Tired and stressed physicians, devastated by the number of deaths and severe, prolonged debilitation of their patients, were less open to stewardship interventions. Difficulty speaking face to face or being present on the wards due to efforts to minimize congestion in work rooms and patient-care areas made communication between antibiotic stewards and treating physicians difficult. ASPs continued to work to improve antibiotic use and to limit overprescribing. ASPs actively monitored those infections, worked to identify emerging outbreaks, and conducted appropriate surveillance to tailor appropriate empiric and definitive antibiotic therapy.

Beyond the essential role of ASPs during the pandemic to reduce the reactive overuse of antibiotics, members of the ASP were involved with assessing emerging treatment regimens for COVID-19; implementing use of remdesivir, dexamethasone, or interleukin-6 inhibitors; and participating in decisions about implementation of monoclonal antibody infusions and vaccinations. The expertise of ASPs in infectious diseases and pharmacologic agents was indispensable across healthcare facilities.^15,20^

Their new stature as CMS-required programs, with the recent pandemic to reinforce their essential value, affords ASPs an opportunity to examine the current approach to ASP structure and implementation. The 2019 annual survey from the CDC National Healthcare Safety Network (NHSN) provides important data to inform this process.^21^

## The 2019 National Healthcare Safety Network Annual Survey

The CMS requires hospitals to report their healthcare-associated infections through the NHSN and conduct an annual survey of infection control practices. In 2014, questions about ASPs were included for the first time. In the most recent 2019 survey, data on ASPs were expanded and made more robust.^21^ The findings provide a snapshot of the increased uptake of ASPs across the country and provide data on the 7 core elements of ASPs: (1) hospital leadership commitment; (2) facility leader(s) accountable for ASP outcomes; (3) pharmacy expertise; (4) action (implementing interventions); (5) tracking the impact of interventions, antibiotic use, and other outcomes such as *C. difficile*; (6) reporting of antibiotic use and resistance; and (7) education for stakeholders.^11^

In 2014, only 40.9% of participating hospitals met all 7 core elements. By 2019, that proportion was 88.9%, and although facility type, bed size, and teaching status varied, ˜80% of critical-access hospitals or hospitals with ≤50 beds met all requirements, an impressive improvement in adoption.^21^ This trend is due in a large part to accreditation and regulatory requirements for an ASP.

Several other findings of interest were identified by this survey. Hospital commitment, as judged by 3 priority commitments (ie, allocation of information technology [IT] resources for ASP efforts and having a physician leader and a pharmacist leader with antibiotic stewardship responsibilities in their contract or job description), is notably more robust for IT support than for stewardship support. Physician and pharmacist steward commitment, as defined by their contract or job description, is as low as 12.9% and 29.6%, respectively, at critical-access hospitals. General acute-care hospitals met this commitment to physician and pharmacy stewards approximately half the time. A 2018 survey suggested that physician steward support of 0.4–1.0 full-time equivalents (FTEs) and pharmacist steward support of 1.0–3.0 FTEs, depending on hospital size (by number of beds), optimizes ASP effectiveness and outcomes.^4^ Adequate support for the stewardship team is essential and should continue to be a focus of advocacy.

The NHSN survey shows that physician and pharmacist stewards co-led ASPs and were jointly accountable for outcomes in >61% of hospitals. Co-leadership was lowest in surgical and critical-access hospitals, with 34.2% and 38.7%, respectively, relying on a pharmacist leader. Physicians are not full-time at many hospitals, including community and rural hospitals. They often have privileges at numerous sites which they visit on a rotating basis. The pharmacist is on site, hired by that facility, and can form deeper relationships with other healthcare providers. Access to pharmacy expertise is high across facilities, ˜95% or greater. By providing that expertise as well as leading or co-leading programs, pharmacy stewards have become the dominant force in ASPs. This evolution has many possible factors.

Infectious diseases expertise in pharmacy has blossomed, with increased numbers of residencies and comprehensive training programs, such as those offered by the Society of Infectious Diseases Pharmacists (SIDP)^22^ or Making a Difference in Infectious Diseases (MAD-ID).^23^ It is unclear whether antibiotic expertise has shown similar growth among physicians and medical students. When medical students were surveyed in 2012, their knowledge of antibiotic stewardship was limited and their knowledge of antibiotics suboptimal.^24^ Physicians who choose to specialize in infectious diseases (ID) receive education about antibiotics, but it is variable. The support that pharmacists and ID-trained pharmacists provide at academic centers, where most ID fellowships are based, can supplant active learning about antibiotics by ID fellows. In qualitative interviews, ID fellows identified the pharmacist steward, and not ID physician leaders, as their primary resource for antibiotic teaching.^25^ Excellent training programs in antibiotic stewardship, developed through the Infectious Diseases Society of America, assume a basic knowledge of antibiotics and focus more on the structure of ASPs, how to conduct interventions in professional and collegial ways, and other aspects of implementation rather than a deep understanding of pharmacology.^26^

Beyond foundational training about antibiotics in school, residency and subspecialty training, the professional activities of pharmacists are more in line with what a steward does compared with physicians. Pharmacists are responsible for verifying and dispensing medications. For all medications, they optimize therapy, review appropriate doses and durations, monitor drug interactions, allergies and adverse events. They track antibiotic resistance and work to contain costs. Indeed, the pharmacy professional approach is one of individual chart review and intervention. In contrast, physicians do not routinely review other physician’s patient care to critique and make recommendations. They have no direct responsibility for verification before the drug is dispensed. They put a high priority on peer respect and collegiality.

Physician time is more costly for hospitals. When stewardship is part of a physician’s contract or job description, the amount of effort assigned and therefore compensation, is often a small percentage. In addition, physicians are more likely to be expected to do a myriad of other activities because their stewardship role is less well defined than that of pharmacist stewards. Pharmacists are more likely to be given a full- or half-time position that allows them to do more intensive stewardship work, form relationships with providers, and be recognized as the hospital antibiotic steward.

However, physicians remain essential to successful programs. They have a deep clinical knowledge and are able to put complicated stewardship decisions into context and understand the mindset of the prescriber. They are able to synthesize the clinical data and judge whether de-escalation is safe or escalation is needed. They can use their clinical expertise to prioritize new initiatives and are often in a better position to advocate with hospital leadership for support and resources. They remain an important resource for the pharmacist steward. In interviews with pharmacist antibiotic stewards, although they do almost all of the day-to-day work, they strongly believe that a physician leader is necessary for an effective program.^27^ A physician who is an ally and active partner was identified as one of the most important components of a successful ASP. Physicians interviewed were similarly supportive of their pharmacist co-lead, and they viewed the co-led team the optimal structure.

Early in antibiotic stewardship, the programs were frequently, though not exclusively, led by physicians. Now that has changed. Although physician groups should focus on the pivotal primary role that physicians play, they must do so recognizing the reality of ASPs today and the leadership of pharmacy. The model that seems most reasonable is recognition that the physician supports their stewardship program with a population health perspective, advocates for new programs and adequate resources, and is available for regular patient review and consultation as needed, while pharmacists lead and perform day-to-day activities.

## Antibiotic stewardship interventions

The NHSN survey also probed interventions to improve antibiotic use. Facility-specific treatment guidelines was a strategy used by >92% of the facilities surveyed while implementation of preauthorization of select antibiotics and prospective audit-and-feedback strategies lagged behind (Table 1). Hospitals with all 3 strategies ranged from 46.3% and 45.9% in children’s and general acute-care hospitals, respectively, to a low of 16.5% in critical-access hospitals. The survey provides little information about the dissemination and implementation of those guidelines. Sites that develop facility-specific guidelines without having a plan to educate, disseminate, and monitor compliance may find that they are fulfilling the letter but not the spirit of that interventional strategy. Guidelines without other strategies such as prospective audit and feedback and/or preauthorization, cited by the ASP implementation guidelines as essential for a robust ASP,^28^ are less likely to be effective.

**Table 1.** Antibiotic Stewardship Program Interventions and the Impact of the COVID-19 Pandemic

Stewardship Intervention	Methods in Practice	Affected by COVID-19?	Going Forward
Pre-authorization	Restricted formulary Approval obtained from AST/ID staff physicians or ID fellows Approval via order form/electronic order if criteria for appropriate use met. If not, further AST/ID approval needed	Increased pushback or pressure to approve agents New disease with high morbidity and mortality Increased workload—process of approval burdensome	Basic AS principles should be adhered to, despite unknowns. Leverage limited approval for up to 24 hours until more clinical/diagnostic data available Expand agents that can be approved via antibiotic order process, verified by any pharmacist, to relieve burden on AST and prescribers
Prospective Audit and Feedback	Identify opportunities to discontinue, de-escalate or escalate therapy, clarify duration of therapy at 48–72 h (or earlier as appropriate) Typically implemented as a voluntary intervention-providers can accept or reject AST recommendations	Overuse of antimicrobials despite no clear bacterial or fungal infection because of inflammatory response associated with COVID-19 (fever, leukocytosis) Providers decline AST recommendations. Less opportunity for face-to-face conversations	Leverage electronic communications and videoconferencing when face-to-face conversations are not feasible. After a process of shared decision making, consider mandatory implementation for a limited number of AST interventions.
Diagnostic stewardship	Reports of microbiology results constructed to maximize stewardship New technologies to improve diagnostics and turnaround time for results Integrate involvement by the AST to interpret and respond to microbiology results	COVID-19 related fever and/or leukocytosis resulted in unnecessary diagnostic testing. Overtesting led to overprescribing for bacterial contaminants or colonization.	Diagnostic stewardship can be utilized to limit inappropriate testing. AST involvement to interpret significance of test results
Duration of Therapy	Define the course of treatment for specific infections based on evidence-based medicine Use of stop dates in antibiotic orders Monitor the full course of therapy from the inpatient setting through hospital discharge	Antibiotic durations prolonged for patients who remained ill despite no evidence of active bacterial or fungal infection	Durations based on diagnosed infection should not be extended without supportive data. When strong evidence (eg, via RCTs) exist for duration, mandatory implementation for a limited number of AST interventions should be considered.
IV to PO optimization	Use of bioavailable oral antibiotics instead of the IV formulation of the same agent Transition to oral from IV antibiotics for susceptible organisms when appropriate	Necessity to shorten lengths of hospital stay because of burden of COVID-19 cases during the surges: oral antibiotics allowed for more timely discharges to home, improved bed availability for acutely ill patients	New data for invasive infections, such as endocarditis or osteomyelitis, suggest equivalent outcomes with oral medications in many scenarios. Optimize opportunities for oral antibiotic therapy
Transitions of care	Improve transition of care from inpatient to outpatient settings for patients on intravenous antibiotics or high-risk oral antibiotics Identify patients being discharged on antibiotics and determine appropriateness of antibiotic choice and duration of treatment	Reduced resources for outpatient visits and monitoring of patients discharged on antibiotics Overwhelmed skilled nursing and long-term care facilities	Established multidisciplinary programs for transitions of care can better withstand unexpected changes in workload. Review of antibiotics prescribed at discharge can identify opportunities for stewardship.
Ambulatory ASP	ASP interventions to reduce inappropriate prescribing in ambulatory care, particularly for acute respiratory tract infections	Increase in telehealth visits may have affected prescribing. In regions with high numbers of COVID-19 cases, prescribing for outpatient respiratory tract infections decreased.	Telemedicine should be further studied for outpatient treatment of common infectious diseases.
Tele-stewardship	Access to stewardship expertise via electronic means—e-mail, phone consultation, or video conferencing Particularly crucial for hospitals that have limited resources and AS expertise, such as rural or critical-access facilities	Increased uptake of telemedicine broadly has potentially opened up new acceptance of telemedicine and made technology more available.	Telemedicine can be an important strategy across settings. Further development in this area can also be useful if there is another surge (eg, with a COVID-19 variant) that makes in-person, ‘handshake stewardship’ challenging.

Note. AST, antibiotic stewardship team; AS, antibiotic stewardship; ID, infectious diseases; RCT, randomized controlled trials; IV, intravenous.

The increased implementation of other ASP strategies is noteworthy and should be the focus of more detailed survey questions in the coming years.

- Diagnostic stewardship has become a major approach. Strategies range from creating customized microbiology reports to guide prescribing and educate prescribers to implementation of new molecular testing with ASP backup.^29^ Implementing new technology can be expensive, but it is worthwhile if it improves evidence-based care. ASP involvement is crucial to guide appropriate use of diagnostic testing and to interpret and apply those tests to optimize antibiotic treatment.^30^
- Each year, more data emerge about the safety of shorter durations of therapy.^31^ Antibiotic therapy for community-acquired pneumonia has evolved from 10–14 day courses to courses as short as 3 days.^32^ Intra-abdominal infections can be treated for 4 days after source control.^33^ Optimizing the duration of therapy is an effective intervention for numerous infections and the shortened exposure to antimicrobials minimizes the selective pressure for antibiotic resistance as well as the risk of *C. difficile* infection.
- Initiatives to transition patients from intravenous to oral antibiotics were originally used to optimize use of bioavailable agents, such as fluoroquinolones, to reduce costs, but they now are transforming the dogma mandating weeks of intravenous therapy for bloodstream infections and endocarditis^34^ or bone and joint infections.^35^ Emerging data of comparable outcomes with oral agents to complete treatment courses for those deep-seated infections are encouraging and provide patients with options that avoid the risk of indwelling lines, limitations on lifestyle, and other disadvantages of long courses of antibiotics.
- Other initiatives include better management at transitions of care.^36,37^ Patients are often discharged on antibiotics that unnecessarily extend durations of treatment. Many patients are also discharged to complete complex antibiotic regimens. ASPs can help coordinate appropriate outpatient monitoring, provide patient education and perform clinical follow-up.
- ASPs are also aggressively moving into the ambulatory arena, both because of accreditation and anticipated regulatory requirements and because of the volume of antibiotics in ambulatory care and high rates of unnecessary use.^38,39^ More than 60% of antibiotic expenditures are in outpatient practices, of which ≥ 30% are inappropriate. Also, 44% of outpatient antibiotic prescriptions are for acute respiratory tract infections, of which half are inappropriate, accounting for 34 million excess prescriptions each year.^40^ Ambulatory pediatric practices have improved inappropriate prescribing because of robust stewardship efforts, but less progress has been seen in adult medicine.
- Antibiotic stewardship via telemedicine is a promising strategy to support healthcare facilities, particularly those without onsite infectious diseases resources such as rural or critical-access hospitals.^41^ The COVID-19 pandemic has necessitated a major shift to telehealth, largely enabled by an unprecedented acceptance of telehealth by insurers, providers, and patients,^42^ and this trend has created an environment that can further promote stewardship via this approach.

## The authority of antibiotic stewardship programs to improve prescribing

These stewardship strategies must be optimally implemented to impact patient care. In qualitative interviews with physician and pharmacist stewards in 2 healthcare systems,^27^ there was strong consensus that stewards should not be ‘antibiotic police.’ Provider engagement strategies are critical to achieve success, and recommendations must be communicated in a collegial manner that does not judge physician competence or autonomy. There must be collaboration, shared decision making, and respect.

But a question that is seldom asked is what if the prescribers are wrong? The time spent convincing recalcitrant providers is significant and can be exhausting and frustrating. ASPs want buy-in and do not want to create conflict. There are countless gray areas in antibiotic stewardship that must rely on the judgment of clinicians providing direct patient care. However, a growing number of strongly evidence-based interventions should not be optional, such as β-lactam antibiotics for methicillin-susceptible staphylococcal infections or short courses of treatment for clinically stable patients with community-acquired pneumonia.

With TJC and CMS support for ASPs, there is explicit acknowledgment that these programs work and are essential for health care. In addition to continued pressure to provide adequate resources for ASPs, serious consideration should be given to advocate for greater enforcement authority by ASPs when interventions are unambiguously supported by clinical experience, research, and guidelines. Although concerns about reducing the collaborative nature of the ASP are valid, we can look to the evolution of infection control programs. There is acceptance that isolation precautions, for example, are not up to the individual providers because they are based on strong scientific evidence and protect the health of other patients and providers. Hospital epidemiologists and infection preventionists remain respected content experts and maintain excellent relationships with other healthcare professionals. ASPs may weaken their own message if those programs lack the conviction to assert that some interventions are so robustly supported by data that an individual provider cannot overrule it.

The next stage in the evolution of ASPs should be the identification of a finite number of stewardship interventions that could be considered mandatory for ASPs and should be set by the CDC as components of its core elements. CMS uses the CDC core elements as the basis of its Conditions of Participation so those interventions would be included in the standards by which the CMS determines whether ASPs are meeting expectations. Such identification would be incorporated into accreditation evaluations. Defined durations of therapy for pneumonia, skin and soft-tissue infections, and urinary tract infections would be logical initial targets for mandatory interventions given the strong evidence base. Those infections are the 3 most common community-onset infections and comprise a significant number of infections treated within health care.^43^ Thus, optimizing the duration of therapy would have a major impact. This approach could serve as an important step toward accomplishing core goals, asserting the strength of the evidence base and the nonoptional nature of the interventions, simultaneously allowing ASPs to focus on difficult discussions with providers about more challenging stewardship decision making.

## Antibiotic stewardship programs and health inequities

Stewardship strives to give the most effective drug with the narrowest spectrum as appropriate for the shortest amount of time in a consistent and equitable manner. There are not enough data about antibiotic prescribing and ASP activities through the lens of equity. On the inpatient side, antibiotic stewards struggle with physicians who have trepidation de-escalating or stopping antibiotics for fear that the patient will relapse or worsen, but we do not typically examine all the factors that influence how providers choose the antibiotic or when they choose to accept ASP interventions. In addition, there has been little examination of the role that bias may play in ASP recommendations. On the outpatient side, physicians often prescribe due to perceived patient demand and to improve patient satisfaction. Studies in ambulatory care have demonstrated that white patients are twice as likely to receive a prescription as African-American patients.^44^ Although many of those prescriptions are inappropriate and therefore African-American patients may be receiving better care, it is for inequitable reasons.

ASPs should commit to examining equity and implicit bias by evaluating their own data and ensuring that ASP recommendations are based on evidence-based medicine and quality care and not influenced by the patient’s race or ethnicity, sex or gender, or other socioeconomic demographics. Professional infectious diseases societies, public health organizations, and other stakeholders should promote examination of these issues. This examination should include discussion and goals related to equity in white papers and guidelines, support for collaborative research in this area, and advocacy for federal funding to study these crucial issues.

In conclusion, antibiotic stewardship has come a long way, but there is a long way to go. With recognition that ASPs are a core function for medical facilities, we can start to reimagine our place in health care. The urgency of success becomes progressively greater as complex patterns of ABR continue to emerge, exacerbated by unpredictable factors such as a worldwide pandemic. Although new agents have been developed, they are not keeping pace with ABR. Those of us participating in ASPs, as well as any provider who writes an antibiotic prescription, have a responsibility to patients and the greater community to make this a high priority.
